# Efficacy and safety of acupuncture combined with Chinese herbal medicine in the treatment of type 2 diabetes mellitus: A protocol for a systematic review and meta-analysis

**DOI:** 10.1097/MD.0000000000027658

**Published:** 2021-10-29

**Authors:** Pengjie Bao, Jia Mi, Ziyang Yu, Le Liu, Zhiyue Zhu, Shilin Liu, Zheng Nan

**Affiliations:** a Changchun University of Chinese Medicine, 1035 Bo Shuo Road, Changchun City, Jilin Province, China; b Endocrinology, First Affiliated Hospital to Changchun University of Chinese Medicine, 1478 Gongnong Road, Changchun City, Jilin Province, China.

**Keywords:** acupuncture, Chinese herbal medicine, meta-analysis, protocol, safety, systematic review, type 2 diabetes mellitus

## Abstract

**Background:**

Diabetes has become a global public health problem and danger to human health. Diabetes is the main cause of blindness, kidney failure, heart attack, stroke, and lower limb amputation. According to the latest epidemiological survey and research, the overall prevalence of diabetes in mainland China is 11.2%, of which type 2 diabetes mellitus (T2DM) accounts for more than 90% acupuncture combined with Chinese herbal medicine have been widely used in the treatment of T2DM. However, we have not found a meta-analysis of their synergistic effects. Therefore, this systematic review and meta-analysis aims to evaluate the efficacy and safety of acupuncture combined with Chinese herbal medicine in the treatment of T2DM.

**Method:**

From inception up to September 20, 2021, the PubMed, Web of Science, Embase, AMED, Cochrane Library, CNKI, VIP, CBM, and Wanfang databases will be searched. The publication date or language will not be limited. We will apply a combination of medical keywords, including “acupuncture”, “Chinese herbal medicine”, and “type 2 diabetes mellitus”. We will also check other ongoing and unpublished studies in the clinical trial registry. At the same time, we will manually search all reference lists from relevant systematic reviews to find other eligible studies. We will use Review Manager software (REVMAN v5.3 Cochrane Collaboration) to meta-analyze the selected literature. The study for acupuncture combined with Chinese herbal medicine in the treatment of T2DM was a randomized controlled study. Two researchers will independently review the research selection, data extraction, and research quality assessments. Finally, we will observe the outcome measures.

**Results:**

This study will generate evidence-based data on the treatment of T2DM with acupuncture combined with Chinese herbal medicine and will provide new ideas and treatment modalities to investigate in future research.

## 1 Introduction

According to the World Health Organization, the prevalence of diabetes is increasing year by year, and its prevalence is rising faster in low- and middle-income countries than in high-income countries. Diabetes is the main cause of blindness, kidney failure, heart attack, stroke, and lower limb amputation. In 2019, it was estimated that diabetes directly caused 1.5 million deaths.^[1]^ According to the latest epidemiological survey and research, the overall prevalence of diabetes in mainland China is 11.2%,^[2]^ of which type 2 diabetes mellitus (T2DM) accounts for more than 90%. Although there are a wealth of drugs and methods for the treatment of diabetes, there are various insulin and oral hypoglycemic agents, such as biguanides, thiazolidinediones, α-glycosidase inhibitors, sodium-glucose cotransporter 2 inhibitors, sulfonylureas, glinide, and dipeptidyl peptidase IV inhibitors, that can effectively lower blood sugar in a short time, eliminate clinical symptoms, and delay the occurrence of complications. However, long-term use of these drugs also leads to drug resistance, and the incidence of diabetes and its complications is still on the rise. Obviously, diabetes has become a global public health problem and danger to human health.^[3]^

In recent years, acupuncture combined with Chinese herbal medicine have been widely used in the treatment of T2DM. As an important part of traditional Chinese medicine, acupuncture can correct various metabolic disorders such as hyperglycemia, overweight, excessive appetite, and hyperlipidemia. Acupuncture can increase insulin sensitivity and improve insulin resistance.^[4,5]^ As a complementary and alternative medicine practice, Chinese herbal medicine has significant advantages in the treatment of diabetes and its complications.^[6,7]^ However, there is no robust evidence showing the effectiveness of acupuncture combined with Chinese herbal medicine in treating T2DM and its side effects; this lack of evidence is also an important factor hindering its promotion in the treatment of T2DM and its complications. Therefore, this systematic review and meta-analysis will evaluate the efficacy and safety of acupuncture combined with Chinese herbal medicine in the treatment of T2DM.

## 2 Materials and methods

### 2.1 Information sources and search strategy

This study will be based on the reporting guidelines for systematic review protocols and meta-analyses (PRISMA-P).^[8]^ This study is a retrospective study and meta-analysis, so the study design, process, and results do not require patient and public participation or ethical approval. From inception up to September 20, 2021, the PubMed, Web of Science, Embase, AMED, Cochrane Library, CNKI, VIP, CBM, and Wanfang databases will be searched. The publication date or language will not be limited. We will apply a combination of medical keywords, including “acupuncture”, “Chinese herbal medicine”, and “type 2 diabetes mellitus”. We will also check other ongoing and unpublished studies in the clinical trial registry. At the same time, we will manually search all reference lists from relevant systematic reviews to find other eligible studies. The expected registration has been approved by the International Platform of Registered Systematic Review and Meta-analysis Protocols. (https://inplasy.com/inplasy-2021-10-0015/). And the registration number is INPLASY2021100015. The search strategy for the PubMed is presented in Table 1.

**Table 1:** Search strategy for the PubMed database.

Number	Terms
#1	Type 2 diabetes mellitus (all field)
#2	Type 2 diabetes (all field)
#3	Diabetic mellitus (all field)
#4	Diabetes (all field)
#5	#1 OR #2-4
#6	Acupuncture (all field)
#7	Needling (all field)
#8	Acupoint (all field)
#9	Acupuncture treatment (all field)
#10	Scalp acupuncture (all field)
#11	Fire needling (all field)
#12	Ear acupuncture (all field)
#13	Intradermal needling (all field)
#14	Auricular acupuncture (all field)
#15	Electroacupuncture (all field)
#16	Catgut embedding (all field)
#17	#6 OR #7-16
#18	Chinese medicine (all field)
#19	Traditional Chinese medicine (all field)
#20	Chinese herb medicine (all field)
#21	Proprietary Chinese medicine (all field)
#22	Chinese Herbs (all field)
#23	Chinese herbal (all field)
#24	#18 OR #19-23
#25	randomized controlled trial (all field)
#26	randomly (all field)
#27	controlled clinical trial (all field)
#28	randomized (all field)
#29	random allocation (all field)
#30	placebo (all field)
#31	single-blind method (all field)
#32	double-blind method (all field)
#33	trials (all field)
#34	comparators
#35	allocation
#36	#25 OR #26-35
#37	#5 And #17 And #24 And #36

### 2.2 Inclusion and exclusion criteria

The inclusion criteria for the literature search will be as follows: studies including patients aged ≥18 years, regardless of sex, with T2DM diagnosed according to the standards of the American Diabetes Association or the World Health Organization; randomized controlled studies; studies in which the experimental group was treated with acupuncture combined with Chinese herbal medicine; studies in which the control group was treated with placebo or hypoglycemic drugs. The exclusion criteria will be as follows: studies including patients aged <18, with non-T2DM; studies in which the experimental group was given treatment methods other than acupuncture combined with Chinese herbal medicine; studies in which the control group was not treated with a placebo or a hypoglycemic drug; studies that were not randomized controlled trials.

### 2.3 Study selection

**Figure 1:**
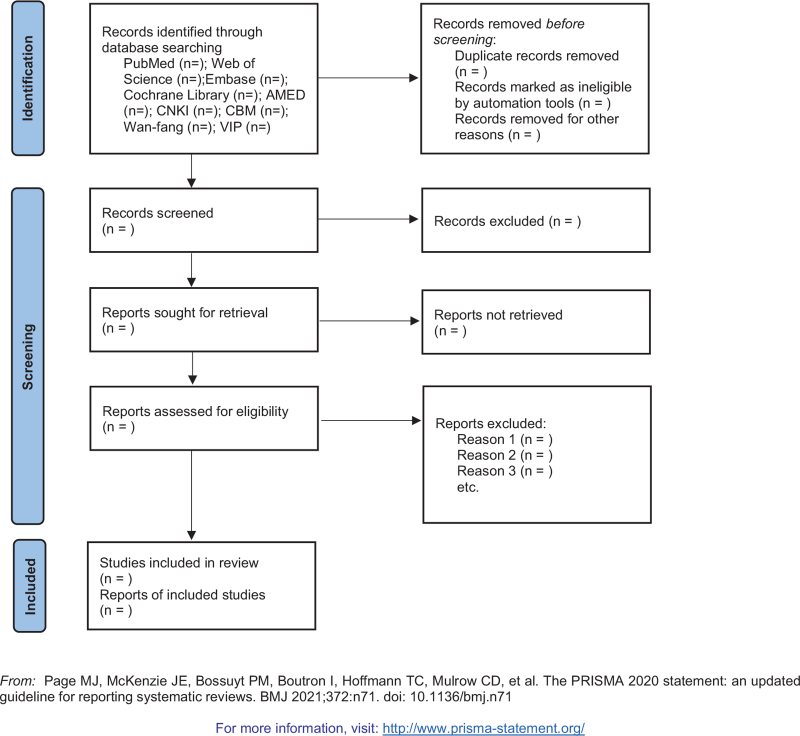
Flow diagram of study selection process.

The 2 researchers will independently carry out the literature search; screening and data extraction will be based on pre-established search strategies, literature inclusion and exclusion criteria, and data extraction tables. We will exclude all conference records, reviews, meta-analyses, newspapers, guides, letters, and other documents. The research selection process will be represented by the PRISMA flowchart.^[9]^ If the full text or the required information on the analytic process is missing from a paper, the author of the study will be contacted, and the data will be requested. The 2 researchers will independently evaluate the methodological quality of the included studies according to the Cochrane manual guidelines, and they will report the results according to the PRISMA guidelines.^[10]^ During the research period, any disagreements between the researchers will be resolved through discussion or negotiation with a third researcher until a consensus is reached. A flowchart of the screening process is presented in Figure 1.

### 2.4 Assessment of study quality

The 2 researchers will use the Cochrane risk bias assessment tool to separately assess the quality of the randomized studies.^[11]^ The assessment content addresses the following: whether the random method is correct; whether the allocation concealment is achieved; whether the blind method is implemented; whether the result data are complete; whether there is selective reporting bias; and whether there are other biases. We will use Begg and Egger tests and set *P* < .1 as statistically significant, and we will use a funnel chart to assess publication bias. If the evaluation quality of any single study is inconsistent between researchers, the discrepancy will be resolved through consensus between the researchers.

### 2.5 Outcome measures

The primary outcome measures will include fasting blood glucose, 2-hour postprandial blood glucose, and glycosylated hemoglobin. The secondary outcome measures will include triglycerides, cholesterol, and insulin secretion. Adverse events resulting from safety violations will be reported.

### 2.6 Statistical analysis

We will use Review Manager software (REVMAN v5.3 Cochrane Collaboration) to meta-analyze the selected literature, and *P* < .05 will be considered statistically significant. The 2 researchers will independently carry out data extraction and input, and a third researcher will conduct inspections, while the first 2 researchers will conduct data calculations. Quantitative data express the combined effect size with standardized mean square error and 95% confidence interval. Binary categorical variables express the combined effect size by odds ratio and 95% confidence interval. The heterogeneity among the included research results will be analyzed by the I^2^ test. If *P* > .05 and I^2^ < 50%, there is homogeneity among the studies, and the fixed-effects model will be used for meta-analysis. If *P* ≤ .05 and I^2^ ≥ 50%, there is heterogeneity among the studies. Sensitivity analysis will then be used to analyze the source of heterogeneity. After the influence of clinical heterogeneity is excluded, the random-effects model will be used for meta-analysis. We will use subgroup analysis based on different interventions, controls, and outcomes.

## 3 Discussion

Diabetes is a global public health problem that affects human physical and mental health, and it is also an independent risk factor for major diseases such as kidney failure, cardiovascular disease, and stroke.^[12,13]^ Although many new drugs for the treatment of T2DM, such as glucagon-like peptide 1 receptor agonists and sodium-glucose cotransporter 2 inhibitors, have been successfully developed, researchers are looking for better blood-glucose-lowering treatment programs. Controlling glucose levels and reducing complications from T2DM involves continual disease management. In the treatment of T2DM, acupuncture combined with Chinese herbal medicine can reduce blood sugar, eliminate clinical symptoms, increase insulin sensitivity, and improve insulin resistance.^[14]^ They can also alleviate the side effects caused by hypoglycemic drugs. Therefore, acupuncture combined with Chinese herbal medicine has broad potential applications in the treatment of T2DM. This study will generate evidence-based data on the treatment of T2DM with acupuncture combined with Chinese herbal medicine and will provide new ideas and treatment modalities to investigate in future research.

## Acknowledgments

All the authors of this manuscript are very grateful to the various departments of Changchun University of Chinese Medicine for their support.

## Author contributions

**Conceptualization:** Pengjie Bao, Zheng Nan.

**Data curation:** Pengjie Bao, Jia Mi, Ziyang Yu.

**Formal analysis:** Le Liu, Zhiyue Zhu.

**Funding acquisition:** Jia Mi.

**Investigation:** Pengjie Bao, Shilin Liu.

**Methodology:** Pengjie Bao, Zheng Nan.

**Project administration:** Jia Mi, Zheng Nan.

**Supervision:** Jia Mi, Zheng Nan.

**Validation:** Le Liu, Zhiyue Zhu, Shilin Liu.

**Visualization:** Pengjie Bao, Jia Mi, Ziyang Yu.

**Writing – original draft:** All authors.

**Final approval of manuscript:** All authors.
